# Viscoelastic Testing for Assessment of Hypercoagulability and Thrombotic Risk in Cancer Patients: A Systematic Review

**DOI:** 10.3390/cancers18213529

**Published:** 2026-11-03

**Authors:** Aleksandra Szymczyk, Franciszek Bałaj, Mariusz Siemiński

**Affiliations:** 1 Department of Emergency Medicine, Medical University of Gdańsk, 80-210 Gdańsk, Poland; mariusz.sieminski@gumed.edu.pl; 2 Pomeranian Medical University in Szczecin, 70-204 Szczecin, Poland

**Keywords:** cancer-associated thrombosis, thromboelastography, rotational thromboelastometry, hypercoagulability, fibrinolysis, systematic review

## Abstract

**Background:** Viscoelastic assays characterize haemostatic abnormalities in cancer, but their clinical prognostic value remains uncertain. We reviewed thromboelastography (TEG), rapid TEG and rotational thromboelastometry (ROTEM) for laboratory phenotyping and thrombotic risk assessment. **Methods:** PubMed/MEDLINE, Embase, Scopus and Web of Science Core Collection were searched through 15 October 2026. During revision, a second reviewer independently verified full-text decisions and extracted data and available quality assessments. Findings were synthesized by temporality, thrombotic endpoint, clinical setting and viscoelastic domain. The protocol was not prospectively registered. **Results:** The review included 64 reports published between 1997 and 2026: 38 grouped with clinical thrombotic endpoints and 26 primarily addressing laboratory findings. Forty-five reports used TEG and 19 used ROTEM. Laboratory abnormalities frequently involved faster clot propagation and increased clot strength, but normal or inconsistent profiles were also reported. Associations with existing thrombosis were distinguished from testing before incident events. High discrimination estimates sometimes belonged to multivariable models, while other studies found little independent prognostic information from TEG/ROTEM. Evidence for fibrinolysis-based prediction was limited and assay-dependent. **Conclusions:** Evidence supports laboratory haemostatic characterization more strongly than individual thrombosis prediction. Current findings do not establish that viscoelastic-guided thromboprophylaxis improves patient outcomes.

## Simple Summary

People with cancer have an increased risk of blood clots, which can cause serious complications. Identifying who will develop a clot remains difficult. This systematic review examined whether thromboelastography and rotational thromboelastometry, tests that measure how whole blood forms and breaks down clots, could help assess this risk. Many studies found that blood from patients with cancer formed clots more quickly, produced stronger clots or showed reduced clot breakdown. However, these laboratory findings did not consistently identify patients who subsequently developed thrombosis. Some promising results came from models combining these tests with other clinical and laboratory information. Current evidence does not establish that using these tests to select preventive blood-thinning treatment improves patient outcomes. Larger, carefully designed studies are needed to determine whether these methods can improve clot risk assessment and guide safer, more personalized care.

## 1. Introduction

Cancer-associated thrombosis is a major cause of morbidity and mortality among patients with malignancy. The risk of venous thromboembolism (VTE), including deep-vein thrombosis (DVT) and pulmonary embolism (PE), is substantially higher in patients with cancer than in the general population and varies according to tumour type, stage, treatment modality, and patient-related characteristics [1]. The pathogenesis of cancer-associated thrombosis (CAT) is multifactorial and involves activation of coagulation pathways by tumour cells, platelet activation, endothelial dysfunction, systemic inflammation, and alterations in fibrinolysis [2]. Despite the clinical importance of thrombotic complications, identifying patients at greatest risk remains challenging. Established risk assessment models, including the Khorana score, provide a framework for risk stratification, but their performance varies across cancer populations and clinical settings [3,4].

Conventional coagulation tests, such as prothrombin time (PT) and activated partial thromboplastin time (aPTT), assess selected components of the coagulation cascade in platelet-poor plasma and primarily measure the time to initial fibrin formation. Consequently, they do not provide a comprehensive assessment of platelet contribution, clot strength or fibrinolysis [5,6]. In contrast, viscoelastic testing methods, including thromboelastography (TEG) and rotational thromboelastometry (ROTEM), provide a dynamic assessment of haemostasis in whole blood by evaluating clot initiation, propagation, clot strength, and fibrinolysis in real time [7,8]. These measurements may therefore provide additional information about the haemostatic abnormalities associated with malignancy.

Several studies have demonstrated hypercoagulable viscoelastic profiles in patients with solid and haematological malignancies, characterized by shortened clotting or clot formation times, increased α-angle, and elevated clot strength or firmness [9,10,11,12]. Similar abnormalities have been reported in myeloproliferative neoplasms and after breast cancer surgery, illustrating the relevance of both the underlying malignancy and the treatment setting to haemostatic assessment [13,14]. However, these findings are not universal: a study of treatment-naïve patients with early breast cancer found no detectable changes in TEG clot kinetics despite microscopic alterations in fibrinogen structure [15].

However, the ability of TEG and ROTEM parameters to predict clinically relevant thromboembolic events remains uncertain, with studies reporting heterogeneous results across different cancer types and clinical settings [4,7,16]. In a prospective study of liver and pancreatic cancer surgery, postoperative pulmonary embolism occurred without preceding thromboelastographic evidence of hypercoagulability [17]. Laboratory evidence of hypercoagulability should therefore be distinguished from the prediction of individual thrombotic events. Likewise, studies comparing patients with and without established thrombosis address a different question from studies in which viscoelastic testing precedes incident events. The performance of multivariable models incorporating viscoelastic parameters alongside clinical characteristics or other biomarkers should also be distinguished from that of TEG or ROTEM parameters alone [18].

Cancer-associated thrombosis may not be explained solely by accelerated clot formation. Impaired fibrinolysis, including study-defined fibrinolysis shutdown, has also been investigated as a potential contributor to thrombotic risk [7,19]. Studies combining viscoelastic testing with complementary haemostatic assays have described impaired fibrinolysis in lymphoma and in patients with colorectal or gastric cancer undergoing chemotherapy [20,21]. These findings support examining clot breakdown alongside clot formation while distinguishing results obtained from viscoelastic assays from those obtained using separate fibrinolysis assays. Their clinical interpretation requires caution because assay methods, definitions, and thresholds differ, and evidence establishing predictive performance for subsequent thrombosis remains limited.

Building on previous reviews [7,22], this systematic review distinguishes laboratory haemostatic phenotypes from associations with existing thrombosis and prediction of subsequent thrombotic events, with particular attention to the timing of viscoelastic measurements. Therefore, the aim of this systematic review was to evaluate the current evidence regarding the use of TEG and ROTEM for the assessment of laboratory hypercoagulability and thrombotic risk in adults with solid and haematological malignancies. We sought to summarize characteristic viscoelastic abnormalities observed in cancer patients, assess their relationships with clinically relevant thrombotic events, and distinguish associations with existing thrombosis from the prediction of subsequent events. Fibrinolysis-related findings were explored as an additional domain within studies identified through the primary hypercoagulability/thrombosis search rather than through a separate comprehensive search targeting fibrinolytic dysfunction. By considering study design, measurement timing, thrombotic endpoints, and individual viscoelastic domains, this review also aimed to identify evidence gaps and priorities for future research.

## 2. Methods

**Figure 1:**
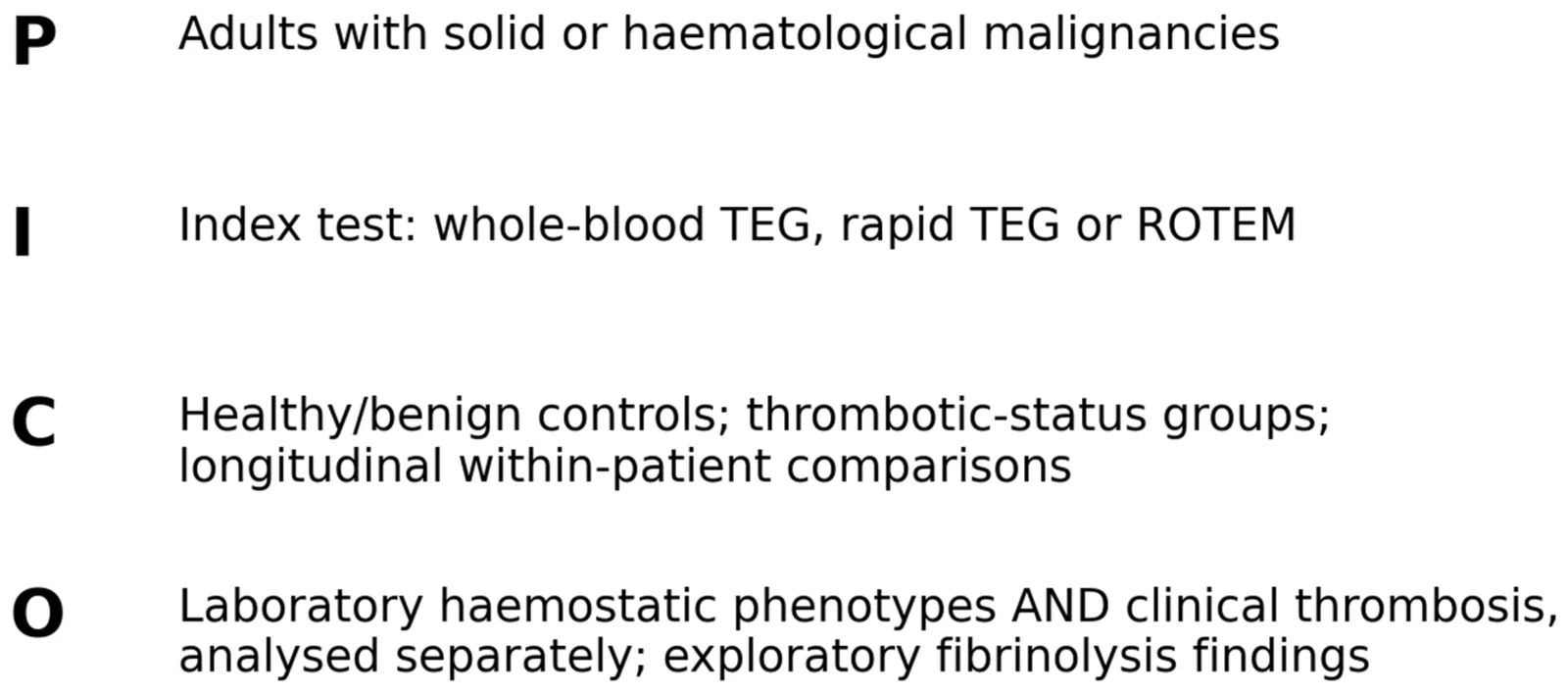
Adapted PICO framework. I denotes the index test rather than a therapeutic intervention. Laboratory phenotypes and clinical thrombotic endpoints are considered separately.

Reporting was guided by PRISMA 2020. The protocol was not prospectively registered. The adapted PICO framework was as follows: population, adults with solid or haematological malignancy; index test, whole-blood TEG, rapid TEG or ROTEM; comparator, healthy or benign-disease controls, patients with different thrombotic status, or longitudinal within-patient measurements; outcomes, laboratory haemostatic phenotypes and clinical thrombotic events (Figure 1). Here, I denotes the index test rather than an assigned therapeutic intervention.

A systematic literature search was performed in PubMed/MEDLINE, Embase, Scopus and Web of Science Core Collection from database inception to 15 October 2026. The search strategy combined controlled vocabulary, where available, with free-text terms related to viscoelastic haemostatic testing, malignancy, thrombosis and hypercoagulability. The PubMed/MEDLINE search strategy was as follows: (“Thrombelastography”[MeSH] OR “rotational thromboelastometry” OR “thromboelastometry” OR “thromboelastography” OR “thrombelastography” OR “TEG” OR “ROTEM” OR “viscoelastic testing”) AND (“Neoplasms”[MeSH] OR “cancer” OR “malignancy” OR “oncology” OR “tumor” OR “neoplasm”) AND (“Venous Thromboembolism”[MeSH] OR “Venous Thrombosis”[MeSH] OR “thrombosis” OR “venous thromboembolism” OR “VTE” OR “pulmonary embolism” OR “DVT” OR “hypercoagulability”). The searches were updated during revision. No restrictions concerning publication date, language or publication type were applied during the electronic searches. Reference lists of eligible studies and relevant reviews were additionally screened to identify potentially eligible publications not retrieved by the database searches. Fibrinolysis-related findings were explored within studies identified by the primary search; no separate comprehensive search for fibrinolytic dysfunction was undertaken. Complete database-specific search strategies, interfaces, search dates and applied limits are provided in Supplementary Table S2.

Original human studies were eligible when they included adults with malignancy and reported extractable whole-blood viscoelastic measurements relevant to haemostatic phenotyping or clinical thrombosis. Laboratory studies were eligible irrespective of whether they demonstrated hypercoagulability; normal or negative findings were retained. Studies assessing incident venous or arterial events, catheter-related thrombosis or portal-vein thrombosis were distinguished from studies without a clinical endpoint. Mixed populations or animal/human studies required separately extractable human cancer data. During revision, eligibility was restricted to full-length articles available in English. Conference abstracts without an eligible full report, reviews, editorials, individual case reports and exclusively animal or in vitro experiments were excluded. Publications devoted solely to tumour diagnosis or staging, without a relevant haemostatic characterization or thrombosis analysis, were outside scope. Treatment studies could contribute eligible baseline or longitudinal haemostatic data, but measurements used solely for treatment monitoring without a relevant review outcome were not sufficient. These refinements were made during revision and were not prospectively registered.

The primary outcomes were viscoelastic evidence of cancer-associated hypercoagulability and objectively confirmed thrombotic events. Hypercoagulability was defined according to study-specific reference ranges or diagnostic criteria and included abnormalities reflecting clot initiation, propagation or strength, such as shortened R, K, clotting time (CT) or clot formation time (CFT), increased α-angle, elevated maximum amplitude (MA) or maximum clot firmness (MCF), and increased study-defined coagulation indices. Relevant thrombotic outcomes included deep-vein thrombosis, pulmonary embolism, catheter-related thrombosis, portal-vein thrombosis and arterial thrombotic events. Secondary outcomes comprised fibrinolysis-related abnormalities, including reduced maximum lysis, increased lysis indices, decreased LY30 and study-defined hypofibrinolysis or fibrinolysis shutdown. The incremental prognostic value of viscoelastic parameters when combined with clinical characteristics, D-dimer concentration, conventional coagulation tests or established thrombotic risk assessment models was also evaluated.

Database records were deduplicated using DOI, PMID and normalized title/year combinations, with manual comparison of titles, authors and journals where needed. Initial screening and extraction were performed by A.S. During revision, F.B. independently verified the full-text inclusion/exclusion decisions and extracted outcome and effect-estimate data and available quality assessments. This was verification during revision rather than a claim that the original search and all screening stages had been independently duplicated from inception. Unavailable reports were distinguished from retrieved reports excluded on eligibility grounds.

Discrepancies were addressed by re-examination of the source publication and discussion between A.S. and F.B.

Initial data extraction was performed by A.S. using a standardized data-extraction form; during revision, F.B. independently verified the extracted outcome and effect-estimate data. Disagreements were resolved by re-examination of the source publication and discussion until consensus was reached between A.S. and F.B. The following information was collected: first author, publication year, country, study design, clinical setting, cancer type and stage, sample size, treatment status, number and type of thrombotic events, duration of follow-up, viscoelastic platform and assay, analyzed TEG or ROTEM parameters, definitions of hypercoagulability, fibrinolysis-related variables, conventional coagulation parameters, statistical methods, effect estimates, predictive performance and principal study findings. Particular attention was paid to parameters reflecting clot initiation, clot propagation, clot strength and fibrinolysis. When information was unclear or incompletely reported, the full text and available Supplementary Materials were examined. Missing or unreported information was recorded as not reported, and no assumptions were made regarding outcomes that were not explicitly presented. Study-level characteristics and extracted findings are presented in Supplementary Table S1A,B. Studies were classified as follows: prospective assessment of incident thrombosis; retrospective prognostic association with testing before the documented outcome; contemporaneous/cross-sectional or case–control assessment of existing thrombosis; or laboratory haemostatic characterization without a clinical thrombotic endpoint. Mixed designs and unclear temporal ordering were identified explicitly. Prospective recruitment alone was not considered evidence that testing preceded thrombosis. The terms prediction and prognostic value were reserved for analyses with sufficiently clear temporal ordering.

Methodological quality was assessed using the Newcastle–Ottawa Scale (NOS), with scores reported separately for selection, comparability, and outcome/exposure, as appropriate to the study design. Where the study design did not permit an appropriate application of the NOS framework used in this review, the assessment was recorded as not applicable (NA) rather than assigning a potentially misleading numerical score.

A structured synthesis without meta-analysis was used, drawing on the transparency principles of SWiM [23]. Evidence was grouped by design and temporality, cancer setting, thrombotic subtype, measurement timing and viscoelastic domain. Effect estimates, uncertainty, event numbers and validation were prioritized over counts of statistically significant studies. Individual-parameter discrimination was kept separate from models combining viscoelastic, clinical and conventional laboratory variables. TEG and ROTEM measurements and cut-offs were not assumed to be interchangeable. A single pooled estimate was not considered clinically interpretable across these different questions. Group comparisons of laboratory values, associations with prevalent events, longitudinal event models and multivariable ROC results address different estimands.

## 3. Results

**Figure 2:**
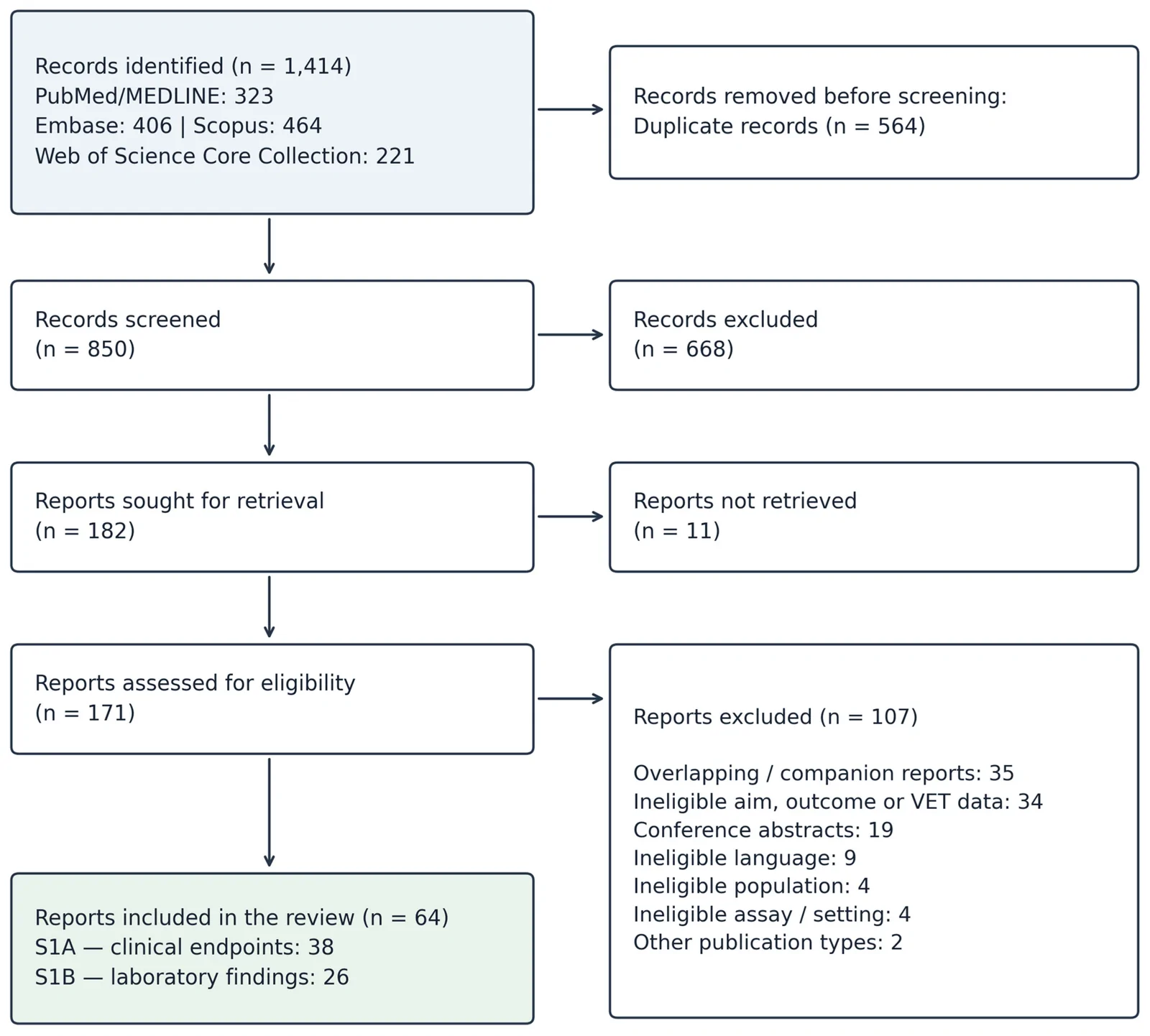
PRISMA 2020 flow diagram of report identification, screening and eligibility assessment. Database searches were completed on 15 October 2026.

The updated database searches, completed on 15 October 2026, identified 1414 records: 323 from PubMed/MEDLINE, 406 from Embase, 464 from Scopus and 221 from Web of Science Core Collection. After removal of 564 duplicate records, 850 records underwent title and abstract screening, of which 668 were excluded. Of 182 reports sought for retrieval, 11 could not be obtained. The remaining 171 reports were assessed for eligibility, and 107 were excluded. A total of 64 reports were included in the review: 38 in Supplementary Table S1A and 26 in Supplementary Table S1B. The selection process and exclusion categories are presented in Figure 2.

The 64 included reports were published between 1997 and 2026. Forty-five used TEG, including rapid TEG where applicable, and 19 used ROTEM. Supplementary Table S1A contains 38 reports grouped with clinical thrombotic endpoints, whereas Supplementary Table S1B contains 26 reports primarily addressing laboratory findings. Twenty-two reports were conducted in China. These are report-level counts and should not be interpreted as the number of independent cohorts. Inclusion in Table S1A does not itself imply that prognostic performance was demonstrated; the temporal relationship between viscoelastic testing and thrombosis and the presence of a direct test–outcome analysis were considered separately.

Longitudinal studies show why laboratory hypercoagulability cannot be equated with clinical prediction. In the full NSCLC cohort, Alexander et al. found no significant prognostic association for individual TEG variables: the subdistribution hazard ratio for MA ≥ 76 mm was 0.8 (95% CI 0.3–2.4). The final model used D-dimer and fibrinogen rather than TEG [24]. Davies et al. likewise did not find significant ROTEM differences between patients with and without subsequent VTE [10].

In postoperative settings, Ahn et al. evaluated preoperative ROTEM against subsequent PE after CRS/HIPEC. Hypercoagulability prevalence differed markedly by definition (39% versus 77%; κ = 0.219); Bayesian analyses supported an association, while frequentist analyses were not statistically significant [25]. Sabharwal et al. reported DVT in 5/31 patients classified as hypercoagulable and 0/21 with normal TEG, but ultrasound was symptom-triggered [26]. Other reports evaluated prostate cancer, bone tumours, colorectal surgery, breast cancer chemotherapy, lung resection and mixed treatment settings [4,16,19,27,28,29,30,31,32]. Their effect estimates, event counts and measurement schedules are presented separately in Table S1A.

Additional studies recorded thrombosis in brain, pancreatic, breast, gastrointestinal or haematological cancer and after surgery for metastatic bone or peritoneal disease [17,21,33,34,35,36,37,38,39,40,41]. These include exploratory cohorts with few events and descriptive event reporting, which do not establish a validated prediction rule. For example, Blasi et al. recorded six events among 27 cholangiocarcinoma patients, but the greater baseline hypercoagulability in those with subsequent events was not statistically significant [37]. De Pietri et al. observed four postoperative PEs without preceding TEG evidence of hypercoagulability [17].

Studies of cancer-associated cerebral infarction assessed patients with an established arterial event. Their TEG differences represent contemporaneous discrimination and cannot be interpreted as prospective stroke prediction [42,43,44]. Cen et al. reported an MA AUC of 0.875 (95% CI 0.819–0.932), but sampling followed stroke onset [42]. Mixed-cancer and lung cancer studies also reported associations with thrombotic status, with temporal interpretation dependent on the actual sampling and diagnostic sequence [36,45].

Studies of gynaecological, lung and gastric cancer reported discrimination based on individual parameters or combinations of TEG with other predictors [18,46,47,48,49]. Their AUC values should not be pooled or ranked without accounting for population, outcome ascertainment, timing and validation. In Gong et al., the combined day-3 TEG model had an apparent AUC of 0.913 (95% CI 0.869–0.957), without independent validation; the temporal ordering of the measurements and DVT diagnosis was insufficiently clear to confirm true prospective prediction [48]. High AUCs from complete models were not attributed to TEG/ROTEM alone.

Venous endpoints predominated, but PE, limb DVT, portal-vein thrombosis, catheter thrombosis and arterial events were clinically distinct. Zanetto et al. specifically examined portal-vein thrombosis in cirrhosis with hepatocellular carcinoma [12]. In cholangiocarcinoma surgery, four portal-vein thromboses and two DVTs were reported [37]. Sun et al. compared patients with prior catheter-device thrombosis with first-implantation patients and additionally followed the latter prospectively; the comparison and follow-up components should remain separate [50].

Gordon et al. recorded two catheter-related internal jugular thromboses in patients with metastatic colorectal cancer but did not provide a VET–event effect estimate [51]. Attaran et al. reported one embolic stroke within a randomized thromboprophylaxis comparison; an isolated event cannot establish predictive performance [52]. The cerebral infarction studies address arterial disease, and their discrimination estimates cannot be transferred directly to VTE prediction [42,43,44]. Where only “VTE” or “TE” was reported, a more specific event type was not inferred.

Laboratory studies in thoracic, digestive, breast, prostate and mixed solid malignancies reported diverse patterns of clot initiation, propagation and strength [9,11,13,15,31,53,54,55,56,57,58,59,60,61,62]. Douroumis et al. related ROTEM findings to clinical risk categories; correlation with a risk score is not evidence of prediction of observed VTE [63]. In early breast cancer, Goncalves et al. did not detect a change in clot kinetics despite microscopic fibrinogen changes [15]. In Laskov et al., the unadjusted α-angle difference was no longer significant after adjustment, and other TEG parameters did not significantly distinguish malignant from benign/premalignant groups [58].

Haematological studies evaluated multiple myeloma, lymphoma, polycythaemia vera and other myeloproliferative neoplasms [14,20,64,65,66,67,68]. Şahin et al. studied 29 untreated MPN patients and 35 healthy controls; EXTEM MCF was 69.7 ± 5.9 versus 58.6 ± 5.3 mm and CFT was 65.3 ± 26.5 versus 109.4 ± 25.5 s (both *p* < 0.01). Clinical thrombosis prediction was not assessed [67]. Crowley et al. found no significant TEG differences between myeloma, MGUS and control groups [66].

Perioperative studies further illustrated that haemostatic profiles can change with surgery and treatment [69,70,71,72,73]. Nates et al. found discordant classifications using the coagulation index and thrombodynamic potential index after craniotomy [73]. During CRS/HIPEC, clot strength could initially decrease, followed by accelerated clot initiation or propagation during HIPEC or increased firmness later in recovery [69,70]. Weinberg et al. demonstrated postoperative biochemical coagulation activation despite frequently normal TEG findings [72]. These observations support physiological characterization rather than a presumption that an abnormal profile invariably predicts thrombosis.

Evidence for the prognostic value of viscoelastic fibrinolysis parameters was limited; two studies reported numerical discrimination estimates for individual fibrinolysis parameters in relation to clinical thrombotic outcomes [19,28]. Tsantes et al. reported INTEM LI60 ≥ 98% as a study-specific shutdown threshold, with an AUC of 0.887 (95% CI 0.824–0.951), sensitivity of 100% and specificity of 67.0% [19]. Bucciol et al. reported LY30 discrimination with an AUC of 0.738, but the reported association did not meet conventional statistical significance (OR 0.421, 95% CI 0.110–1.04; *p* = 0.062) [28]. Aleksandrowicz et al. described impaired fibrinolysis and later events but did not estimate formal predictive performance [21]. These reports should not be conflated with studies that simply included a lysis parameter in the assay panel.

Laboratory evidence of reduced fibrinolysis was described in myeloma and other haematological malignancies [20,65]. In Bønløkke et al., the principal clot–lysis assessment was a separate dynamic clot formation/lysis assay, while ROTEM characterized clot formation and strength; the lysis findings should not be attributed automatically to ROTEM [20]. Hypofibrinolysis, study-defined shutdown and abnormal values in different assays were not treated as equivalent. External validation and clinically transferable cut-offs remain insufficiently established.

The evidence includes small cohorts, descriptive series, retrospective comparisons and model development studies with limited event counts. Important limitations concern participant selection, clinical confounding, ascertainment of thrombosis and uncertain temporal ordering. Aggregate NOS totals alone do not establish low risk of bias for prediction models.

## 4. Discussion

In this systematic review, viscoelastic testing identified frequent, but not universal, hypercoagulable phenotypes across a wide spectrum of solid and haematological malignancies. This pattern was observed in studies involving lung, gastrointestinal, pancreatic, colorectal, breast, prostate, hepatocellular and gynaecological cancers, as well as multiple myeloma, despite considerable differences in study populations, clinical settings and viscoelastic platforms [9,10,12,34,46,54,55,56,65]. Across TEG studies, the hypercoagulable phenotype was most commonly characterized by shortened R and K times, increased α-angle and elevated MA [9,34,55,56]. In studies using ROTEM, corresponding abnormalities included shortened CT or CFT, increased α-angle and elevated MCF [10,11,12,65]. Collectively, these abnormalities indicate accelerated clot initiation and propagation together with increased clot strength. However, no individual TEG or ROTEM parameter was consistently abnormal across all malignancies, suggesting that cancer-associated hypercoagulability represents a multidimensional haemostatic phenotype rather than an abnormality captured by a single assay variable. Laboratory phenotypes, associations with existing thrombosis and assessment of incident events were therefore interpreted separately. Several studies demonstrated associations between selected viscoelastic parameters and objectively confirmed thrombosis, particularly parameters related to clot strength and clot propagation [46,47]. By contrast, other investigations reported inconsistent associations or limited predictive performance despite detecting hypercoagulable viscoelastic profiles [4,10]. Overall, these findings suggest that the current evidence is considerably stronger for the identification of cancer-associated hypercoagulability than for the prediction of individual thrombotic events, supporting the interpretation of viscoelastic testing as a potentially complementary rather than standalone component of thrombotic risk assessment [8,22].

A recurring observation in this review was the consistency of findings among studies primarily designed to characterize laboratory evidence of hypercoagulability rather than to predict subsequent thrombotic outcomes. The expanded evidence base includes studies with normal or unchanged viscoelastic findings as well as studies reporting hypercoagulability [15,58,66,73]. Importantly, however, the viscoelastic phenotype was not uniform or static. Hypercoagulability persisted during remission in patients with multiple myeloma, changed during the perioperative period in patients with brain tumours, and varied with treatment in colorectal and prostate cancer [9,33,56,64]. These observations suggest that viscoelastic abnormalities may reflect the combined influence of tumour biology, disease burden, treatment exposure and the clinical setting rather than a fixed haemostatic signature of malignancy. Notably, Khatri et al. and Mou et al. observed viscoelastic changes consistent with a hypercoagulable phenotype despite PT and aPTT remaining within their respective reference ranges. These findings suggest that conventional plasma-based coagulation tests may not reflect relative or dynamic haemostatic changes detected by whole-blood viscoelastic assays [33,34]. Selected studies additionally suggested abnormalities of clot breakdown, which are discussed separately below. Nevertheless, because these studies were not specifically designed or adequately powered to evaluate subsequent thrombotic events, their findings primarily support the biological characterization of cancer-associated hypercoagulability rather than its clinical prognostic application.

The revised evidence base includes both older investigations identified during reassessment and more recently published reports. Many additional reports describe laboratory haemostatic phenotypes rather than validated prediction of clinical thrombosis. The expanded evidence base therefore broadens the characterization of cancer-associated coagulation changes without establishing a corresponding increase in evidence for individual thrombotic risk prediction.

While cancer-associated hypercoagulability was commonly identified, the evidence supporting the prognostic value of viscoelastic testing was considerably more heterogeneous. Studies with different temporal structures evaluated objectively confirmed clinical thrombotic outcomes, including catheter-related thrombosis, postoperative venous thromboembolism, pulmonary embolism, portal-vein thrombosis and cancer-associated arterial ischaemic events. Rather than relying solely on comparisons of individual viscoelastic parameters between patients with and without thrombosis, several studies used multivariable analyses, receiver operating characteristic analyses or prediction models to assess the contribution of TEG or ROTEM findings to thrombotic risk assessment [42,46,47]. However, these studies did not converge on a single universal marker. Context-specific associations were reported for parameters reflecting clot initiation (R), propagation (K), clot strength (MA and MCF), and composite coagulation indices [12,42,46,47]. Some studies also suggested that serial or postoperative measurements may provide more information than a single baseline assessment, indicating that the timing of viscoelastic testing may influence its prognostic performance [27,47]. In addition to parameters of clot formation and strength, one study suggested that fibrinolysis-related variables may also provide prognostic information [19]. Nevertheless, some of the highest reported discriminatory values were obtained from models combining viscoelastic parameters with clinical or laboratory variables rather than from TEG or ROTEM alone. Their performance should therefore not be attributed exclusively to viscoelastic testing. Overall, these findings suggest that the potential clinical value of TEG and ROTEM lies in providing complementary and context-dependent prognostic information rather than identifying a single parameter applicable across all malignancies. Associations with established cerebral infarction or catheter thrombosis cannot establish pre-event prediction. Prospective recruitment, ROC analysis or a high AUC does not itself demonstrate that the test preceded the outcome. Validation and calibration must also be considered.

Other studies questioned the ability of individual viscoelastic parameters to reliably predict thrombotic events. Importantly, the reasons for uncertain prognostic evidence differed between studies. Davies et al. demonstrated a hypercoagulable ROTEM profile in patients with lung cancer, but the measured parameters did not distinguish patients who subsequently developed VTE [10]. In other studies, individual TEG parameters showed limited discriminatory performance, lost significance after adjustment for established risk factors, or were inferior to D-dimer or clinical prediction models [16,28,29,30]. Several smaller or primarily exploratory studies detected perioperative, treatment-related or chemotherapy-associated changes in viscoelastic profiles but included too few thrombotic events, or were not designed appropriately, to establish reliable prognostic performance [4,31,53]. Thus, some inconclusive findings may reflect limited statistical power, differences in measurement timing and heterogeneity of clinical endpoints rather than the complete absence of an association between viscoelastic abnormalities and thrombosis. Moreover, most studies focused predominantly on parameters of clot initiation, propagation and strength, whereas fibrinolysis-related variables were inconsistently assessed or were not evaluated as prespecified predictors. Consequently, the limited predictive performance of conventional TEG or ROTEM parameters does not exclude the possibility that hypofibrinolytic phenotypes, including fibrinolysis shutdown, may provide additional prognostic information. However, this possibility currently remains insufficiently investigated. Collectively, these findings suggest that the major source of heterogeneity in the available literature concerns the translation of group-level laboratory hypercoagulability into clinically meaningful prediction of individual thrombosis. A hypercoagulable viscoelastic profile should therefore not automatically be interpreted as evidence of increased individual thrombotic risk, and the available evidence suggests a potential complementary role in risk assessment that requires validation rather than an established standalone predictive application.

Fibrinolytic dysfunction emerged as a potentially important but insufficiently investigated component of cancer-associated hypercoagulability. Although several studies included fibrinolysis-related variables such as LY30, maximum lysis, LI45 or LI60 among the recorded viscoelastic parameters, these variables were rarely evaluated as prespecified predictors of clinical thrombosis [4,10,29,42,43]. Direct observations were reported in studies in patients with multiple myeloma and bone tumours. Treliński et al. demonstrated a combined hypercoagulable and hypofibrinolytic ROTEM profile in multiple myeloma, characterized by increased FIBTEM clot firmness, reduced maximum lysis and elevated LI45 and LI60 [65]. In patients undergoing surgery for bone tumours, Tsantes et al. found that increased LI60 was associated with postoperative thromboembolic complications and reported prognostic information from fibrinolysis shutdown, defined in that study as INTEM LI60 ≥ 98%, as the strongest evaluated prognostic marker, with greater discriminatory performance than D-dimer in that cohort [19]. It is important, however, to distinguish hypofibrinolysis, which broadly describes reduced fibrinolytic activity, from fibrinolysis shutdown, which represents a more extreme phenotype defined using assay-specific thresholds and measurement time points. Taken together, these findings suggest that the prothrombotic haemostatic phenotype in malignancy may result not only from accelerated clot formation or increased clot strength but also from the prolonged persistence of clots due to impaired breakdown. This interpretation is consistent with recent reviews identifying impaired fibrinolysis as a potentially important but underexplored mechanism of cancer-associated thrombosis [7,22]. Additional evidence from patients with haematological malignancies suggests that impaired fibrinolysis may coexist with increased clot strength; in Bønløkke et al., however, clot lysis was evaluated using a separate dynamic assay rather than inferred from ROTEM alone [20]. Nevertheless, the prognostic significance of fibrinolysis-related parameters remains preliminary because only a small number of studies have directly linked them to clinical thrombotic outcomes, and the proposed thresholds have not undergone independent external validation. Aleksandrowicz et al. (2026) also described impaired fibrinolysis in gastrointestinal cancer but did not establish formal predictive performance. These observations were identified through the primary thrombosis/hypercoagulability search, which may have missed studies focused specifically on fibrinolysis [21].

The findings of this review have several important clinical implications. The consistent identification of hypercoagulable viscoelastic profiles across diverse malignancies suggests that TEG and ROTEM may provide information on global haemostatic status that is not fully captured by conventional plasma-based coagulation tests [33,34]. However, the available evidence does not support their use as standalone tools for predicting individual thrombotic events or guiding thromboprophylaxis. Some of the highest reported discrimination estimates were observed when viscoelastic parameters were incorporated into multivariable models or interpreted alongside established clinical and laboratory risk factors, including D-dimer and tumour-related characteristics [16,47]. Conversely, several studies found that individual TEG parameters provided limited additional discrimination or performed less well than D-dimer or established clinical risk models [16,28,29]. These findings suggest that the greatest potential of viscoelastic testing lies in complementing rather than replacing existing risk assessment strategies. Fibrinolysis-related parameters may provide additional information in selected populations, but their clinical applicability remains preliminary and requires prospective validation [19]. Importantly, no included study demonstrated that thromboprophylaxis guided by TEG or ROTEM reduced the incidence of thrombosis or improved other clinical outcomes. Viscoelastic testing should therefore be considered a potential adjunct requiring validation rather than an independent decision-making tool for thromboprophylaxis, an interpretation consistent with recent reviews of its potential application in oncology [7,22].

Several limitations of the available evidence should be considered when interpreting the findings of this review. Considerable clinical and methodological heterogeneity existed with respect to cancer type, disease stage, treatment status, study population, viscoelastic platform, assay parameters and clinical endpoints. The included outcomes encompassed VTE (including deep-vein thrombosis and pulmonary embolism), catheter-related thrombosis, portal-vein thrombosis and cancer-associated arterial ischaemic events, which represent clinically and mechanistically distinct complications. Many studies included few thrombotic events, limiting precision and increasing the risk of overfitting in multivariable models. Twenty-two of the 64 reports in the current inventory were conducted in China, while several cancer types were represented by only one or two studies, potentially limiting the generalizability of the findings. Substantial variability was also observed in the timing of viscoelastic testing, including preoperative, postoperative, perioperative and treatment-related measurements, as well as in thromboprophylaxis exposure, definitions of hypercoagulability and cut-off values used to classify abnormal results. These differences prevented meaningful quantitative synthesis and may partly explain the inconsistent prognostic findings. External validation of proposed parameters, cut-offs and prediction models was generally absent. Finally, although several studies recorded fibrinolysis-related variables, only a few systematically evaluated impaired fibrinolysis as a prespecified predictor or directly linked it to clinical thrombotic outcomes. The original single-reviewer workflow and absence of prospective registration remain review-level limitations. Independent verification during revision reduces errors but does not retrospectively constitute fully duplicated initial screening. English-language eligibility and revision-stage refinements may introduce additional selection limitations. The absence of a dedicated PROBAST assessment limits our appraisal of the risk of bias in the reported prediction models.

Future research should prioritize prospective, multicentre and cancer-specific studies using standardized viscoelastic protocols and objectively confirmed clinical endpoints. Measurement time points should be prespecified in relation to surgery, systemic treatment and thromboprophylaxis, and longitudinal testing should be considered to determine whether temporal changes provide greater prognostic information than a single baseline measurement. Venous, arterial, catheter-related and portal-vein thrombotic events should be analyzed separately. Candidate viscoelastic parameters should be evaluated against established clinical risk models and biomarkers such as D-dimer, with reporting of discrimination, calibration, incremental predictive value and clinical utility. Models combining TEG or ROTEM with clinical and laboratory variables require independent external validation before routine implementation. Particular attention should be given to fibrinolysis-related parameters, including the standardization of definitions and cut-off values for hypofibrinolysis and fibrinolysis shutdown. Ultimately, interventional studies would be required to determine whether viscoelastic-guided risk assessment or thromboprophylaxis improves patient outcomes without producing an unacceptable increase in bleeding.

## 5. Conclusions

Viscoelastic testing commonly identifies a hypercoagulable phenotype across a broad spectrum of solid and haematological malignancies. However, current evidence is considerably stronger for the characterization of cancer-associated haemostatic abnormalities than for the prediction of individual thrombotic events and does not support TEG or ROTEM as standalone predictive tools. Their most plausible clinical role is as an adjunct to established clinical and laboratory risk assessment strategies, particularly within cancer-specific multivariable models. Fibrinolysis-related parameters, including fibrinolysis shutdown, represent a potentially important but insufficiently validated component of thrombotic risk assessment. Prospective multicentre studies using standardized protocols, prespecified cut-offs and objectively confirmed outcomes are needed to evaluate incremental predictive value and determine whether viscoelastic-guided management improves clinical outcomes.

## Data Availability

Extracted study-level data are provided in Supplementary Table S1A,B. The primary publications are identified in the reference list.

## Supplementary Materials

The following supporting information can be downloaded at: https://www.mdpi.com/article/10.3390/cancers18213529/s1, Table S1A reports grouped with clinical thrombotic endpoints; Table S1B reports primarily characterizing laboratory haemostatic findings; Table S2 reports complete database-specific search strategies, interfaces, search dates and applied limits.
