# Supplementary material for: Viscoelastic Testing for Assessment of Hypercoagulability and Thrombotic Risk in Cancer Patients: A Systematic Review
Source: Cancers (Basel). 2026 Nov 3;18(21):3529. doi: 10.3390/cancers18213529 (PMC13762158; doi:10.3390/cancers18213529)
Supplement: Supplementary file 1 [file cancers-18-03529-s001.zip › cancers-4634505-supplementary Table S2.pdf]

Supplementary Table S2 Complete database specific search strategies

Manuscript ID cancers 4634505

Viscoelastic Testing for Assessment of Hypercoagulability and Thrombotic Risk in Cancer Patients: A Systematic Review

The electronic searches were rerun from database inception to 15 October 2026. No restrictions concerning publication date, language or publication type were applied. The exact database specific strategies, platforms and numbers of records retrieved are reported below.

Search overview

Database	Platform or interface	Final search date	Records
PubMed MEDLINE	PubMed	15 October 2026	323
Embase	Embase.com	15 October 2026	406
Scopus	Scopus Advanced Search	15 October 2026	464
Web of Science Core Collection	Web of Science Core Collection (Clarivate)	15 October 2026	221

Total records before deduplication: 1,414.

Complete strategies

S2.1 PubMed MEDLINE

Database: PubMed MEDLINE
Platform or interface: PubMed
Final search date: 15 October 2026
Records retrieved: 323

Exact search syntax

("Thrombelastography"[MeSH] OR "rotational thromboelastometry" OR "thromboelastometry" OR "thromboelastography" OR "thrombelastography" OR "TEG" OR "ROTEM" OR "viscoelastic testing") AND ("Neoplasms"[MeSH] OR "cancer" OR "malignancy" OR "oncology" OR "tumor" OR "neoplasm") AND ("Venous Thromboembolism"[MeSH] OR "Venous Thrombosis"[MeSH] OR "thrombosis" OR "venous thromboembolism" OR "VTE" OR "pulmonary embolism" OR "DVT" OR "hypercoagulability")

Search limits and notes: No date, language or publication type filters were applied.

S2.2 Embase

Database: Embase
Platform or interface: Embase.com
Final search date: 15 October 2026
Records retrieved: 406

Exact search syntax

(thromboelastography:ti,ab,kw OR thrombelastography:ti,ab,kw OR thromboelastometry:ti,ab,kw OR 'rotational thromboelastometry':ti,ab,kw OR TEG:ti,ab,kw OR ROTEM:ti,ab,kw OR 'viscoelastic testing':ti,ab,kw) AND (cancer*:ti,ab,kw OR malignan*:ti,ab,kw OR oncolog*:ti,ab,kw OR tumor*:ti,ab,kw OR tumour*:ti,ab,kw OR neoplasm*:ti,ab,kw) AND (thrombosis:ti,ab,kw OR

thromboembol*:ti,ab,kw OR 'venous thrombosis':ti,ab,kw OR 'venous thromboembolism':ti,ab,kw OR VTE:ti,ab,kw OR 'pulmonary embolism':ti,ab,kw OR DVT:ti,ab,kw OR hypercoagulab*:ti,ab,kw)

Search limits and notes: No date, language or publication type filters were applied. The export comprised 380 bibliographic records and 26 clinical trial registry records.

S2.3 Scopus

Database: Scopus
Platform or interface: Scopus Advanced Search
Final search date: 15 October 2026
Records retrieved: 464

Exact search syntax

TITLE-ABS-KEY(thromboelastography OR thrombelastography OR thromboelastometry OR "rotational thromboelastometry" OR TEG OR ROTEM OR "viscoelastic testing") AND TITLE-ABS-KEY(cancer* OR malignan* OR oncolog* OR tumor* OR tumour* OR neoplasm*) AND TITLE-ABS-KEY(thrombosis OR thromboembol* OR "venous thrombosis" OR "venous thromboembolism" OR VTE OR "pulmonary embolism" OR DVT OR hypercoagulab*)

Search limits and notes: No date, language or publication type filters were applied.

S2.4 Web of Science Core Collection

Database: Web of Science Core Collection
Platform or interface: Web of Science Core Collection (Clarivate)
Final search date: 15 October 2026
Records retrieved: 221

Exact search syntax

TS=(thromboelastography OR thrombelastography OR thromboelastometry OR "rotational thromboelastometry" OR TEG OR ROTEM OR "viscoelastic testing") AND TS=(cancer* OR malignan* OR oncolog* OR tumor* OR tumour* OR neoplasm*) AND TS=(thrombosis OR thromboembol* OR "venous thrombosis" OR "venous thromboembolism" OR VTE OR "pulmonary embolism" OR DVT OR hypercoagulab*)

Search limits and notes: No date, language or publication type filters were applied.

Eligibility restrictions were applied during study selection rather than through database filters. Fibrinolysis-related findings were explored within the studies retrieved by this primary thrombosis/hypercoagulability search; a separate comprehensive search specifically targeting fibrinolytic dysfunction was not performed.
